# The Complexities of Assessing Language and Interpreter Preferences in Pediatrics

**DOI:** 10.1089/heq.2017.0057

**Published:** 2018-05-01

**Authors:** Maya I. Ragavan, John D. Cowden

**Affiliations:** ^1^ Department of Pediatrics, Boston Medical Center, Boston, Massachusetts.; ^2^ Department of Pediatrics, Children's Mercy Kansas City, Kansas City, Missouri.

**Keywords:** family-centered care, health disparities, interpreter, language, pediatrics

## Abstract

Providing patients, parents, and families high-quality healthcare in the language of their choice is a fundamental component of patient-centered care in pediatric settings. However, language needs may be complex and dynamic, creating clinical and ethical challenges in cases of provider–parent discordance regarding the need for an interpreter. In this perspectives article, we use a clinical encounter as a foundation to discuss the intricacies of addressing language needs in pediatrics. We also describe the urgent need for further innovation and improvement in linguistic supports available to diverse patients and families.

## Introduction

Before starting a clinical visit at a pediatric primary care clinic, I (M.I.R.) asked a patient's mother about her language needs and she requested we speak in English. As she began an intricate story about her child's health, it became clear that there were language barriers. It was challenging for her to explain her child's symptoms in what was her second (or maybe third) language and equally difficult for me to fully comprehend the nuances of her story as I did not speak her first language. Thinking I was empathizing with her, I stopped her a few times to ask if she would like to use an interpreter. She eventually became frustrated and said, “Do you think my English is bad!? I have been practicing and attending classes.” We called an interpreter, but I could sense she was upset. After the visit, I reflected on how language impacts the parent–provider relationship in all pediatric healthcare settings and my role as a pediatrician to best support the language needs of patients, parents, and families.

The immigrant population is growing exponentially, comprising 13.5% of the U.S. population in 2016.^1^ Of those who are foreign-born, 49% (around 21 million) have limited English proficiency.^1^ Conducting clinical encounters in a patient's language of choice is essential, as language is used to paint a picture of the patient's health, ensure high-quality and safe care, discuss diagnosis and management, and build a trusting rapport.^2,3^ However, as highlighted in the above vignette, language preference is complex and can be contextualized within the larger dynamics of acculturation. Contemporary models of acculturation describe a multidimensional process of potential changes in cultural practices, values, and identities.^4^ Language preference for immigrants, like other aspects of acculturation, is often dynamic rather than binary.^4^ Patients may learn English, stop using some of their native language, or speak different languages depending on the context. This can be even more complicated for visits with older children and adolescents, whose language preferences and acculturation patterns may differ from those of their parents.^5^

When a provider and patient (or parent) disagree about the need for an interpreter, legal and ethical tensions can arise. As described in a National Academy of Medicine report, patients have the right to choose whether to use interpreter services.^3^ However, to promote patient safety, it is the provider's responsibility to ensure the patient fully understands the diagnosis and care plan.^6^ This conflict between patient-centered care and patient safety creates challenging questions. Is it patient-centered care to use an interpreter against a patient's wishes? On the other hand, is it patient-centered care if the patient does not fully understand the clinical encounter? Furthermore, is it the right of the provider to determine how much English is “enough” to conduct the visit without an interpreter?

To explore these tensions, it is important to consider patients' perspectives about interpreters. Extant literature has described the importance of professional interpreters and provider-perceived barriers that may interfere with interpreter use in clinical settings.^2,3,7,8^ Less research has focused on reasons patients may choose not to use an interpreter.^9^ A few studies have described patients' concerns about interpreters, including breeches of confidentiality, lack of interpreter availability, poor-quality interpretations, discrimination related to low English proficiency, and adding time to the visit.^10,11^ Patients also may think that interpreters change the dynamics of the visit and impede the development of provider–patient rapport.^12^ A National Academy of Medicine report describes anecdotes of adult patients who choose to speak in English to accommodate their English-speaking providers; the authors go on to state that no studies have determined how frequently this occurs.^3^

Another important consideration is the type of interpreter used during clinical visits. Although professional interpreters are recommended to minimize errors and promote patient safety, studies have shown that a variety of *ad hoc* interpreters may be used in clinical settings, including front desk staff, members of the medical team, and accompanying family members.^2,13–15^ There may be provider–patient discrepancies in choice of interpreter, for example, two studies found that patients may prefer using family members as interpreters.^11,15^

In the pediatric setting, children of immigrants, who often are more likely than their parents to speak English comfortably,^5^ may be asked to interpret, putting them in the role of “language broker.”^16^ Asking children to be language brokers is problematic because they are unlikely to fully understand both languages, may have conflicts of interest, and lack proper training in professional interpretation.^2^ This raises the risk of miscommunication and medical error. Furthermore, language brokering is associated with children experiencing stress, anxiety, and depression.^16–18^ In contrast, recent qualitative studies have described positive aspects of language brokering, such as a closer connection between a child and their family, improved child self-esteem, a sense of pride for parents and their children, and an opportunity for children to stay fluent in their parent's native language.^16,18^ Nevertheless, current best practice holds that children should never be asked to interpret during clinical encounters due to the high risk of incomplete communication and poor outcomes.

Providers must also carefully consider the way they relay their own language skills to patients. Studies have highlighted potential benefits of using bilingual providers, including patients' preference for speaking directly with their providers,^11^ equal quality of care between bilingual providers and interpreters,^19^ and worse patient perceptions of providers who are language discordant compared with bilingual.^20^ Therefore, it may be important for bilingual providers to give patients the opportunity to choose between using an interpreter and speaking directly with the provider. However, the definition of what constitutes a bilingual provider lacks standardization and often relies only on providers' self-reported proficiency. Past work has shown that pediatric providers at academic institutions conducted visits without interpreters even if they did not rate their Spanish language skills as proficient.^21^ A study from the adult literature reported similar findings, describing that physicians conduct visits in their second language, even when limited in fluency, to save time, for convenience, and as a chance to improve their language skills.^22^ Therefore, provider competency in languages other than English should be seen as a formally taught and tested professional skill, replacing the traditional approach that has allowed and even encouraged providers to just “get by” with unmeasured language proficiency.^23^

In recent decades, the field of medicine has made significant strides in addressing the need for professional interpreters. To understand the complex and dynamic ways language and culture affect the patient–provider relationship, providers must progress beyond simply asking “do we need an interpreter for this visit?” The next step is learning how to explore the language preferences of everyone involved in a pediatric clinical encounter, including the parent, the child, other family members, and each member of the healthcare team.

## Implications for Pediatric Healthcare

First, we must consider how we ask about language. The National Academy of Medicine suggests asking at least two language questions in the healthcare setting, one focused on the patient's English proficiency and the other on the patient's preferred language during clinical encounters.^3^ In addition to these questions, it may be helpful to ask the parent, child (if age appropriate), and other family members about their language preferences to determine how to conduct the visit in a language that is appropriate for everyone the parent wishes to include. Clinics and hospitals should also consider providing information before the visit (e.g., in the waiting room) on the availability and potential costs of interpreter services, so families can make an informed decision regarding interpreter use. In settings receiving federal funds, families may not know that interpretation must be provided free of charge. It also could be useful to update parents' language preferences at each visit in the patient's electronic medical record, just as we do with allergies and medications.

Second, education for healthcare providers and interpreters on how to best recognize and address parents' potential concerns about interpreter services is necessary to ensure families receive culturally sensitive care. Providers should be trained in handling complex scenarios involving interpreters. Examples include when parents and their older children have different language preferences, when parents request interpreters during only portions of a visit, and when parents prefer to use interpreters only with specific members of the healthcare team. Providers should consider the impact of language brokering on child wellness and the parent–child relationship, as well as how to engage families in conversations about the risks and benefits of language brokering. For example, if a parent would like her child to interpret, providers may wish to use a professional interpreter, while finding another way to engage the child in the visit to foster pride in the child's language abilities. To assure that providers do not exacerbate issues of language discordance by using inadequate language skills, institutions can provide clear expectations through polices, language testing, and provider trainings regarding who is allowed to provide care in non-English languages.

Finally, we must continue to support the unique needs of immigrant families. The American Academy of Pediatrics (www.aap.org/en-us/about-the-aap/Committees-Councils-Sections/Council-on-Community-Pediatrics/Pages/Immigrant-Child-Health-Toolkit.aspx) has discussed the value in creating safe spaces for families to talk about their experience immigrating to and living in the United States. Those conversations may help illuminate how language and cultural identities shift over time. In addition, further attention should be given to addressing potential parent–child cultural differences (e.g., differences in language preferences) during clinical settings. Cultural brokers, who can translate both linguistic terms and cultural worldviews, have been used to bridge language and cultural divides.^24^ Such brokers can support immigrant families by mediating cultural conflicts and language differences that may arise within the parent–child–provider triad.

The concern I felt for the mother who preferred not to use an interpreter, but was not able to fully communicate with me, has led me to ask parents detailed questions about their language preferences and explain more about the interpreter service programs at the clinic. In some cases, asking these questions helped address potential communication barriers; in others, the complexity of their reasons required more unpacking than time allowed. Every case was a powerful reminder that despite medicine being rooted in the need to dichotomize and neatly categorize, the patient–provider relationship always will require us to recognize subtle shades of gray.
